# Contraceptive Prevalence and Consulting Service in Women with Systemic Lupus Erythematosus: A Cross-Sectional Study

**DOI:** 10.4314/ejhs.v31i2.12

**Published:** 2021-03

**Authors:** Maryam Mobini, Reza Ali Mohammadpour, Yasaman Salehi, Fatemeh Niksolat

**Affiliations:** 1 Diabetes Research Center, Mazandaran University of Medical Sciences, Sari, Iran; 2 Department of Biostatistics, Diabetes Research Center, Faculty of Health, Mazandaran University of Medical Sciences, Sari, Iran; 3 Student Research Committee, Faculty of Medicine, Mazandaran University of Medical Sciences, Sari, Iran; 4 Orthopedic Research Center, Department of Rheumatology, Mazandaran University of Medical Sciences, Sari, Iran

**Keywords:** Lupus Erythematosus, Systemic, Contraception, Counseling, Pregnancy, Family Planning Services

## Abstract

**Background:**

Systemic lupus erythematosus (SLE), often affects women of childbearing age. Family planning consultation is a major aspect of medical care in these patients because of the risk of disease activation and poor pregnancy and fetal outcomes. The aim of the present study was to evaluate contraceptive prevalence and consulting service in women with SLE.

**Methods:**

In a cross-sectional study, a total of 144 female patients with SLE, ages 15–50, who were presented to rheumatology clinics in Sari, north of Iran, were evaluated. The study was conducted between March 2019 and May 2020. Patients' clinico-demographic profile and fertility information were obtained. Disease activity and damage were assessed by the systemic lupus erythematosus disease activity (SLEDAI) and Systemic Lupus International Collaborating Clinics/American College of Rheumatology (SLICC/ACR) Damage Index (SDI).

**Results:**

One hundred and forty-four SLE patients of childbearing age participated in this study. From 102 patients with the possibility of pregnancy, 36(35.2%) received contraceptive consultations in last year. Withdrawal was the most prevalent contraceptive method (41.7%), followed by permanent (11.8%), and barrier methods (9%). There were no significant differences in age, disease duration, marriage duration, SDI or SLEDAI scores between the women who received or not received contraceptive counseling (P>0.05).

**Conclusion:**

Many SLE patients did not receive adequate information about contraception, and it may be associated with many adverse effects on disease activity and pregnancy outcomes. Therefore, contraceptive consultation as an important aspect of patient's management is strongly suggested.

## Introduction

Systemic lupus erythematosus (SLE), as a multisystem autoimmune disorder, often affects women of childbearing age (1). Some women with SLE plan for pregnancy and some do not have access to appropriate methods of contraception. Considering that these patients are of childbearing potential, they are at risk for unplanned conception. Unplanned pregnancies women with SLE can have serious consequences for both mother and child. The risk of disease relapse is increased to 20% during pregnancy. Also, in women with SLE, pregnancy is associated with increased risk of adverse fetal and maternal outcomes, including fetal loss, prematurity, low birth weight, congenital heart block, postpartum hemorrhage, and need to blood transfusion (2–4). Therefore, family planning and contraception are especially important in women with SLE and should be explained to patients soon after the diagnosis of SLE. It is believed that using oral contraceptives in SLE patients who are in active phase of disease is associated with an increased risk of thrombosis, especially in patients with the presence of dyslipidemia, obesity, hypertension, smoking and positive antiphospholipid antibodies (5–6). Also, major flares may occur in women with SLE on combined oral contraceptives (7). Hormonal contraception can be used in patients with inactive disease and low risk of thrombosis (1). It has been shown that progestogen-only contraceptives are not associated with an increased risk of disease recurrence (5–6). Additionally, using the copper intrauterine device (IUD) is not believed to be associated with worsening disease activity or infection in women with SLE (5).

Despite the crucial importance of contraception in women with SLE, it has been revealed that most of women who are at increased risk for unplanned pregnancy, do not receive appropriate contraceptive counseling (8–10). Therefore, these women will benefit from careful contraceptive counseling prior to pregnancy which can potentially lead to better maternalfetal outcomes. The present study was designed to investigate the contraceptive prevalence and consulting service in women with SLE.

## Methods

**Study design and sample**: This cross-sectional study was performed in patients with SLE at rheumatology clinics in Sari, north of Iran from March 2019 to May 2020. A total of 144 patients with SLE were evaluated.

**Inclusion and exclusion criteria**: Married female patients, aged between 15–50 years, with a confirmed diagnosis of SLE according to the Systemic Lupus International Collaborating Clinics classification criteria (SLICC) (11) without considering pregnancy and disease activity were included. Patients' mental disability or inability to be interviewed were considered as exclusion criteria.

**Data collection:** Demographic and fertility information, and clinical features related to SLE such as age, level of education, place of residence, duration of marriage, current contraceptive method, number of previous pregnancies, previous pregnancy complications, history of spontaneous abortion, disease duration, involvement of the major systems, presence of antiphospholipid antibodies and drug history were recorded in eligible patients.

History of major systemic manifestations including hematologic involvement (hemolytic anemia, platelets below 100,000 or severe leukopenia), lupus nephritis class 3–5, cardiac involvement (myocarditis lupus), lung involvement, and central nervous system manifestations (history of stroke, transverse myelitis, cerebral vasculitis, psychosis, and seizure) were recorded. A history of thrombotic events, including stroke, heart attack, deep vein thrombosis, pulmonary embolism, and retinal vein thrombosis, was also obtained from the patients. Disease activity and damage were assessed by the systemic lupus erythematosus disease activity (SLEDAI) and Systemic Lupus International Collaborating Clinics/American College of Rheumatology (SLICC/ACR) Damage Index (SDI), respectively (12–13).

Pregnancy planning was categorized based on this question: Which of the following correctly describes your condition over the last three months? 1. I was trying to get pregnant, 2. No matter if I am pregnant, 3. I used contraception, and, 4. This question does not apply to me (without sexual contact, permanent method of contraception, including vasectomy in the partner or tubal ligation in patient, or being pregnant or menopause). In patients at risk of pregnancy, contraceptive methods over the last three months (combination pill, progesterone pill, condom, IUD, rhythm, withdrawal and permanent methods) were questioned. Also, fertility history including pregnancy outcomes (number of live births, premature miscarriage during the first trimester, late miscarriage, stillbirth, ectopic pregnancy, and induced miscarriage) and menopausal status were assessed.

**Sample size calculation:** The estimation of sample size was based on a study by Yazdany et al., who reported that 59% of SLE patients received no contraceptive counseling (8); statistical power of 80%, and a type I error of 5%, with the formula for calculation of sample of prevalence studies.

**Ethical consideration:** This study was approved by the Vice Chancellor for Research and Technology and Ethics Community of Mazandaran University of Medical Sciences (IR.MAZUMS.IMAMHOSPITAL.REC.1398.50 89). Patients or their companions were explained about the aim of the study and informed consent was obtained.

**Statistical analysis:** The data's normal distribution was assessed using Kolmogorov-Smirnov statistical test. Mean and standard deviation (SD) as well as frequency (%) were used to display quantitative and qualitative data. Also, chi-squared, student's t-test or Mann-Whitney U t-test were used for data analysis. P value less than 0.05 was considered statistically significant.

## Results

The mean of age and disease duration were 35.55± 8.3(16–49 years) and 5.96± 5.2 (three months-33 years), respectively. The mean of SLEDAI and SDI scores were 1.07±0.3 and 0.31±0.9, respectively. Three (2.1%) of the patients experienced thrombotic events, 54(37.5%) were found with one or more major organ involvement, 31 were eligible for pregnancy, and 22(70.9%) used withdrawal method. Demographic and clinical data of patients with SLE were shown in Table 1.

**Table 1:** Demographic and clinical features of patients with SLE

Variables		Mean±SD N(%)	
**Age**; years (Mean±SD)		35.55± 8.3	
**Urban residency** (Number, %)		110 (76.4%)	
**Level of education** (Number, %)	Illiterate	2 (1.4%)	
	Primary	6 (4.2%)	
	Secondary	82 (56.9%)	
	College	54 (37.5%)	
**Disease duration**; years(Mean±SD)		5.96± 5.2	
**Major organ involvement** (Number, %)	Hematologic	31(21.5%)	54 (37.5%) patients had one or more major organ involvement
	Lupus nephritis	25(17.4%)	
	Cardiac	3(2.1%)	
	Lung	1(0.7%)	
	Central nervous system	11(7.6%)	
**Antiphospholipid antibodies** (Number, %)		23(16%)	
**Medication treatment** (Number, %)	Prednisolone	134(93.1%)	4 patients (2.8%) received rituximab and 6 (4.2%) used cyclosporine/tacroli mus.
	Hydroxychloroquine	125(86.8%)	
	Azathioprine	38(26.4%)	
	Methotrexate	16(11.1%)	
	Mycophenolate mofetil	21(14.6%)	

Among the study population, 107(74.3%) were married, 31(21.5%) were single, and 6(4.2%) were widowed or divorced. Five patients were menopause and from 102 remained married patients, 36(35.2%) received contraceptive consultations in the last year. There were four pregnant women in the study. The statistical analysis on contraception methods was done on 98 eligible patients. Seventy-four (75.5%) women used contraceptive methods in the last three months and withdrawal was the most prevalent method of contraception, reported by 60(61.2%) patients. Fertility condition and contraception methods of patients with SLE are shown in Table 2.

**Table 2:** Fertility condition and contraception methods of patients with SLE

Variables		Mean±SD N(%)	
**Marriage duration**; years (Mean±SD)		14.60± 8.1	
**In last three months** (Number, %)	Trying to get pregnant	11 (7.6%)	
	No matter if pregnant	3 (2.1%)	
	Used a contraceptive method	74 (51.3%)	
	Does not apply to the patient	56 (38.9%)	
**Contraceptive methods in** **eligible patients (n=98)** (Number, %)	OCP	1(1.0%)	6 patients tend to be pregnant.
	IUD	1(1.0%)	
	Withdrawal	60(61.2%)	
	Condom	13(13.2%)	
	Permanent methods	17(17.3%)	
**Total pregnancy** (Number± SD)	1.85±1.1		

OCP: Oral contraceptive pill, IUD: intrauterine device

Thirty patients had 46 pregnancies after SLE diagnosis. Pregnancies outcome included 21 live birth, 13 early abortion, one late abortion and seven induced abortion. From 54 patients with major systemic involvement, 13(24%) had pregnancy (19 pregnancies), one of them was pregnant during the study, 8 with live children (44.4%), 5 with early abortion, one with late abortion and 4 with inductive abortion. There were no significant differences in age, disease duration, marriage duration, SDI or SLEDAI scores between the women who received or not received contraceptive counseling (P>0.05).

## Discussion

The present study demonstrates that about two-thirds of SLE patients with pregnancy risk, did not receive adequate counseling about contraception in the last year. And, 74.4% of the patients used contraceptive methods over the last three months. The most common contraceptive method practiced by patients was withdrawal. This method has a high failure rate (14). Other commonly used methods were permanent and barrier methods.

In the current study, 35.2% of the patients with SLE received contraceptive consultations in the last year which shows a relatively lower rate compared to other studies (8,9,15,16). A study in Brazil found that among 85 women with SLE, 53% did not use any contraceptive method but 22% used condoms, 11% used combined oral contraceptives and 7% used hormone injections (9). In another study in Turkey, 20.3% of SLE patients did not use any contraceptive methods, but using withdrawal and condom were found to be more common (withdrawal 32.7%, condom 28.3%) (16). According to Cravioto et al. discontinuation rate for different methods of contraception is significantly different; 35% in OCP, 55% in progestin-only pills (POP), and 29% in IUD (17). In a study in Thailand, among the SLE patients at risk for unintended pregnancy, only one-third used effective contraceptive methods and one-fifth did not have any background knowledge about SLE and pregnancy (10). Yazdany et al. also observed that among 206 women with SLE, 86 were at risk for unplanned pregnancy, of whom 22% were inconsistent contraceptive users, 53% depended solely on barrier methods and IUDs were used by 13% (8). In Galappatthy et al's. study live birth after SLE diagnosis was estimated to be 45% and adverse fetal outcomes (fetal loss, pre-maturity, low birth weight) were significantly more prevalent in SLE patients (2). In the present investigation, five of 11 married patients were using mycophenolate mofetil, and all three patients on rituximab received consultation about contraception in the last year. In a study in 206 women with SLE, those using potentially teratogenic medications reported not receiving contraceptive counseling, not using contraception consistently, or not using more effective contraceptives (8). In a study in the USA, one-third of 68 SLE patients, did not receive contraception counseling when starting new medications. Older age, white race, depressive symptoms, and higher SLE disease activity were independently associated with not receiving contraception counseling (18).

Women with positive antiphospholipid antibodies are not good candidates for OCP, because of elevated risk of thrombosis (5). Use of OCP should be limited to patients with nonsevere and stable disease, without thrombosis risk factors (6). In an open trial on eight patients, six received OCP and two were allocated to the control group. During a 12-month follow-up, three patients among the OCP users experienced four major flares, but the two patients in the control group were found with no disease exacerbation (7).

In the current study, OCP was used by three SLE patients that in one patient was associated with disease flare (lupus nephritis). From 23(15.9%) patients who were positive for antiphospholipid Ab/lupus anticoagulant, 14 patients were married, of whom 12 used withdrawal and only two participants were on barrier methods of contraception and tubal ligation, and none received OCP.

According to the present findings, contraception consultation seems to be ignored in many SLE patients while many do not have a reliable method for contraception. Despite low rate of contraception consultation, SLE patients prefer to avoid pregnancy which could be due to their knowledge or experiences; nevertheless, social and economic issues, including the cost of the disease, also may play a role.

Our study has some limitations. First, this study was conducted in a single center in the north of Iran and the results may not be generalizable to women's with SLE in other geographic regions, particularly those with different cultural contexts. Second, the incomplete information on gynecologic history could be a potential confounding factor and a limitation of our study.

In conclusion, our findings seem to be useful in improving contraceptive counseling in women with SLE. Many SLE patients did not receive adequate information about birth control methods, and it may be associated with many adverse effects on disease activity and pregnancy outcomes. According to the current study, a multidisciplinary team approach is needed to improve patient knowledge about SLE and pregnancy, and relatedly contraceptive consultation.
